# The comprehensive complication index as a tool for reporting the burden of complications after mini-percutaneous nephrolithotomy: is it time to leave the Clavien–Dindo classification behind?

**DOI:** 10.1007/s00345-022-04045-9

**Published:** 2022-05-28

**Authors:** Luca Boeri, Matteo Turetti, Carlo Silvani, Irene Fulgheri, Letizia Maria Ippolita Jannello, Susanna Garbagnati, Matteo Malfatto, Gilda Galbiati, Efrem Pozzi, Stefano Paolo Zanetti, Fabrizio Longo, Elisa De Lorenzis, Giancarlo Albo, Andrea Salonia, Emanuele Montanari

**Affiliations:** 1 Department of Urology, IRCCS Foundation Ca’ Granda, Ospedale Maggiore Policlinico, Via della Commenda 15, 20122 Milan, Italy; 2 Department of Vascular Surgery, Foundation IRCCS Ca’ Granda, Ospedale Maggiore Policlinico, Milan, Italy; 3 Department of Clinical Sciences and Community Health, University of Milan, Milan, Italy; 4 Division of Experimental Oncology/Unit of Urology, URI, IRCCS Ospedale San Raffaele, Milan, Italy

**Keywords:** Percutaneous nephrolithotomy, Complications, Clavien–Dindo classification, Comprehensive complication index, Stone

## Abstract

**Purpose:**

To validate the comprehensive complication index (CCI) for mini-percutaneous nephrolithotomy (mPCNL).

**Methods:**

Data from 287 patients who underwent mPCNL were analyzed. Complications after mPCNL were classified using both the CCI and the Clavien–Dindo classification (CDC). Descriptive statistics and linear/logistic regression analyses detailed the association between clinical predictors and mPCNL outcomes.

**Results:**

After mPCNL, 83 (28.9%) patients had complications, of which 12 (4.2%) patients with multiple complications had a higher CCI score compared to the traditional CDC system accounting only for the highest grade. The CCI enabled a more accurate prediction of length of stay (LOS) than CDC (CCI: *r* = 0.32; *p* < 0.01 vs. CDC: *r* = 0.26; *p* = 0.01). Patients with multiple complications had higher stone volume (*p* = 0.02), longer operative time and LOS (all *p* < 0.01). A higher rate of post-operative hospital readmission (33.3% vs. 9.9%, *p* = 0.02) and lower rate of stone free (33.3% vs. 64.7%, *p* = 0.04) were found in patients with multiple complications than in those with single complication. Linear regression analysis revealed that multiple complications were associated with longer LOS (*p* < 0.001) after accounting for BMI and stone volume. Similarly, having multiple complications was associated with fivefold higher risk of readmission (*p* = 0.02).

**Conclusion:**

The CCI is a valuable metric for assessing post-operative complications after mPCNL. The cumulative CCI is a better predictor of LOS than the CDC for mPCNL. Minor complications not captured by the highest CDC score are relevant since patients with multiple complications have longer LOS and higher rate of readmission than those with single ones.

**Supplementary Information:**

The online version contains supplementary material available at 10.1007/s00345-022-04045-9.

## Introduction

Percutaneous nephrolithotomy (PCNL) is the gold standard surgical technique for large renal stones in adult patients [1]. Various studies have shown that PCNL is a highly successful procedure [2], but it can be associated with serious complications, including post-operative fever, pain, bleeding requiring blood transfusion, pneumothorax and infectious complications [3, 4]. In recent years, the miniaturization of the instruments and the adoption of new technologies have decreased the rate of treatment-related complications while maintaining good outcomes. Mini-PCNL (mPCNL) was found to be equally safe and effective than standard procedure but with lower blood loss and transfusion rates [5]; moreover, PCNL with vacuum-assisted devices were associated with lower operative time, hospitalization costs and infectious complications than classic mPCNL [6, 7].

In clinical practice, the Clavien–Dindo classification (CDC) [8] is the most popular system for assessing perioperative morbidity and mortality. The European Association of Urology recommends the systematic application of the CDC in urology [9] in order to improve complication scoring associated with different urological procedures [10–13]. However, the CDC has several limitations that limit its readability and interpretation. For instance, the absence of a weighting system restricts cross-grade comparison, thus precluding the comparison of a patient with three grade I complications vs. one grade III complication. Additionally, most studies report only the highest-grade complication, leading to data loss and underestimation of the total burden of complications in patients who have both low- and high-grade adverse events [6, 14, 15].

To this aim, the Comprehensive Complication Index (CCI) has been recently introduced in clinical practice to score the burden of post-operative adverse events [16]. The CCI is based on the CDC but accounts for all accumulated complications and provides a continuous overall score. This novel tool was introduced in general surgery [17] but has also been validated for major urological and endourological procedures [16, 18, 19]. Kowalewski et al., investigated the application of the CCI in radical cystectomy, radical prostatectomy and partial nephrectomy series and they found that this novel tool was more accurate than the CDC in reporting the true complication burden [16]. Similarly, Grüne et al. found that the CCI captured the cumulative morbidity of PCNL and ureteroscopy (URS) more accurately than the CDC [19]. Despite its acceptance for major uro-oncological surgery, the CCI has been poorly investigated for minimally invasive PCNL.

In particular, we wanted to introduce the CCI in mPCNL, which is the most widely employed PCNL option by the urological community [20]. Therefore, in this study, we aimed: (i) to validate the CCI for mPCNL and (ii) to assess the clinical value of the CCI over the CDC in terms of surgical outcomes.

## Materials and methods

We performed a retrospective analysis of all consecutive patients who underwent mPCNL for renal stones at a single tertiary-referral academic center between January 2017 and December 2021. Mini-PCNL represented the technique of choice when PCNL was planned, except in case of complete or almost complete staghorn stones, for which standard PCNL (24 Ch) was indicated.

Patient’s demographics were collected. Health significant comorbidities were scored with the Charlson comorbidity index (CHI), further segregated as 0 vs. ≥ 1 [21]. Weight and height were measured for each participant and body mass index (BMI), defined as weight in kilograms by height in square meters, was calculated. A pre-operative urographic computed tomography (CT) scan was requested in each patient, which was used for the estimation of stone density (Hounsfield unit-HU) [22] and stone location. The stone volume was calculated using the ellipsoid formula (length  ×  width  ×  height  ×  π × 1/6) [23, 24].

All procedures were performed by two experienced (> 150 PCNL performed) endourologists (E.M; F.L.) in a standardized fashion as previously described [6, 25].

Briefly, with the patient placed in the supine Valdivia position, under general anesthesia, a ureteral catheter was placed in the renal pelvis to inject contrast medium. Subsequently, the renal puncture was performed with combined fluoroscopic/ultrasonographic control and tract dilation was executed one-shot with a 16 Ch metallic dilator. A 16 Ch metallic sheath was placed and the 12 Ch Karl Storz “minimally invasive PCNL” (MIP) nephroscope was introduced [25]. A 550 μm Holmium:YAG laser fiber (VersaPulse PowerSuite 100 W, Lumenis, Israel) was used for stone fragmentation; laser setting was adapted according to surgical needs. After lithotripsy, a 8 Ch nephrostomy tube was positioned as exit strategy, while the ureteral catheter was removed in all cases.

Number of the percutaneous tracts and operative time (OT) were recorded. The evaluated post-operative data included hemoglobin drop and length of hospital stay (LOS). According to internal protocol of our institution, uncomplicated procedures were managed as follows: on post-operative day one the bladder catheter was removed and the nephrostomy tube was closed; on post-operative day two a percutaneous pyelography was performed to assess ureteral canalization and the presence of residual stones. If ureteral canalization was confirmed, the nephrostomy tube was removed. Patients were discharged on post-operative day three.

**Fig. 1:**
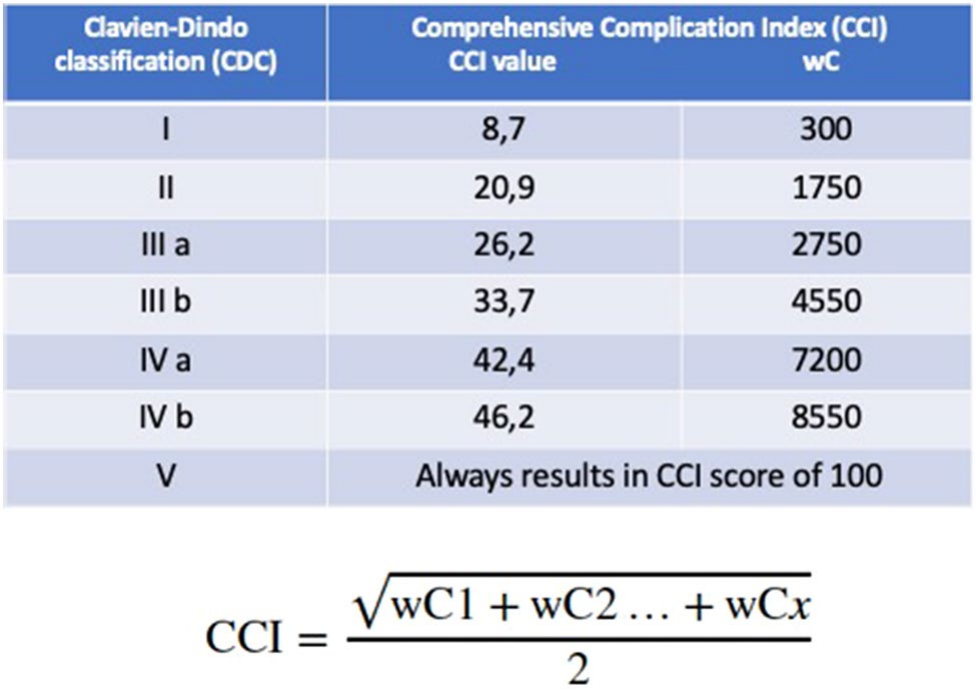
Calculation of the Comprehensive Complication Index

Only events that occurred during the hospital stay or within 30 days of the initial procedure were considered. Post-operative complications were graded according to the PCNL-adjusted Clavien Score (CDC) [10]. For the specific purpose of this study, for every patient we recorded each single complication with its severity (as for CDC). Furthermore, after listing all complications for each patient, the CCI score was generated using the freely available online tool www.assesssurgery.com [16]. Of note, each of the Clavien grades is assigned a specific CCI value and weight of complication (wC) (Fig. 1); finally, the overall CCI score is calculated with the formula reported in Fig. 1.

Patient counseling and follow-up were standardized among the cohort. All patients were instructed to return to the emergency department of the same hospital if they developed post-PCNL complications. Phone calls for the evaluation of possible complications were scheduled 15 and 30 days after surgery as per standard clinical protocol. Patients were evaluated within 3 months after surgery with CT scan to identify residual stones. The stone-free (SF) rate was defined as the absence of residual fragments. Patients with residual fragments were offered, according to stone dimension, observation or auxiliary procedures including second-look PCNL, extracorporeal shockwave lithotripsy, or retrograde intrarenal surgery.

We excluded patients with congenital renal anomalies (*N* = 15); scheduled staged procedures for large stone burden (*N* = 27); concomitant additional procedures other than PCNL (*N* = 15); endoscopic combined intrarenal surgery procedures (*N* = 2). A final cohort of 287 patients who underwent mPCNL for kidney stones was considered for statistical analysis.

Data collection adhere to the principles of the Declaration of Helsinki. All patients signed an informed consent agreeing to share their own anonymous information for future studies. The study was approved by the Foundation IRCCS Ca’ Granda—Ospedale Maggiore Policlinico Ethical Committee (Prot. 25508).

### Statistical analysis

Distribution of data was tested with the Shapiro–Wilk test. Data are presented as medians (interquartile range; IQR) or frequencies (proportions). Descriptive statistics were used to describe the whole cohort. Clinical parameters, intraoperative and post-operative characteristics were compared among patients with single vs. multiple post-operative complications with the Mann–Whitney test and Fisher Exact Test, as indicated.

Univariable and multivariable linear regression models were used to investigate potential predictors of hospitalization time. Similarly, logistic regression models tested the association between clinical variables and re-hospitalization event in the whole cohort. Statistical analyses were performed using SPSS v.26 (IBM Corp., Armonk, NY, USA). All tests were two sided, and statistical significance level was determined at *p* < 0.05.

## Results

Table 1 details demographic characteristics of the whole cohort. Overall, median (IQR) age and BMI were 56 (47–65) years and 24.6 (22.0–27.7) kg/m^2^, respectively. A CHI ≥ 1 was found in 105 (36.6%) participants. Above all, 191 (66.6%) patients had multiple stones, with a median stone volume of 2.2 (1.0–4.6) cm^3^. Multiple access tracts were used in 49 (17.1%) cases. Median operative time and LOS were 107 (80–140) min and 4 (3–6) days, respectively.

**Table 1:** Demographic characteristics of the whole cohort (*n* = 287)

Age (years)	
Median (IQR)	56 (47–65)
Range	19–84
Male Gender [No. (%)]	175 (61.0)
BMI (kg/m^2^)	
Median (IQR)	24.6 (22.0–27.7)
Range	17.9–46.1
CHI (score)	
Median (IQR)	0.0 (0.0)
Mean (SD)	0.6 (0.2)
Range	0–6
CHI ≥ 1 [No. (%)]	105 (36.6)
Laterality [No. (%)]	
Right	137 (47.7)
Left	150 (52.3)
Stone volume (cm^3^)	
Median (IQR)	2.2 (1.0–4.6)
Range	0.5–26.3
Multiple stones [No. (%)]	191 (66.6)
Stone density (Hounsfield unit)	
Median (IQR)	1280 (880–1423)
Range	100–2286
Multiple access tracts [No. (%)]	49 (17.1)
Operative time (min)	
Median (IQR)	107 (80–140)
Range	36–255
Hospitalization time (days)	
Median (IQR)	4 (3–6)
Range	2–22
Hemoglobin drop (g/dL)	
Median (IQR)	1.3 (0.6–2.1)
Range	0.0–6.0
Post-operative complications [No. (%)] (Highest Clavien score)	
Clavien–Dindo I	24 (8.4)
Clavien–Dindo II	45 (15.7)
Clavien–Dindo IIIa	10 (3.5)
Clavien–Dindo IIIb	4 (1.4)
Multiple complications [No. (%)]	12 (4.2)
Post-operative complications [No. (%)] (Overall number)	
Clavien–Dindo I	36 (12.5)
Clavien–Dindo II	50 (17.4)
Clavien–Dindo IIIa	10 (3.5)
Clavien–Dindo IIIb	4 (1.4)
Comprehensive complication index (score)	
Median (IQR)	20.9 (8.6–20.9)
Range	8.6–34.8
Stone-free rate [No. (%)]	219 (76.3)

*BMI* body mass index, *CHI* Charlson Comorbidity Index

Of 287, 83 (28.9%) patients had post-operative complications, of which 24 (8.4%), 45 (15.7%), 10 (3.5%) and 4 (1.4%) were of highest CDC of I, II, IIIa and IIIb, respectively. Supplementary Table 1 reports the type and the severity of post-operative complications. Median CCI score was 20.9 (8.6–20.9) (Table 1).

**Fig. 2:**
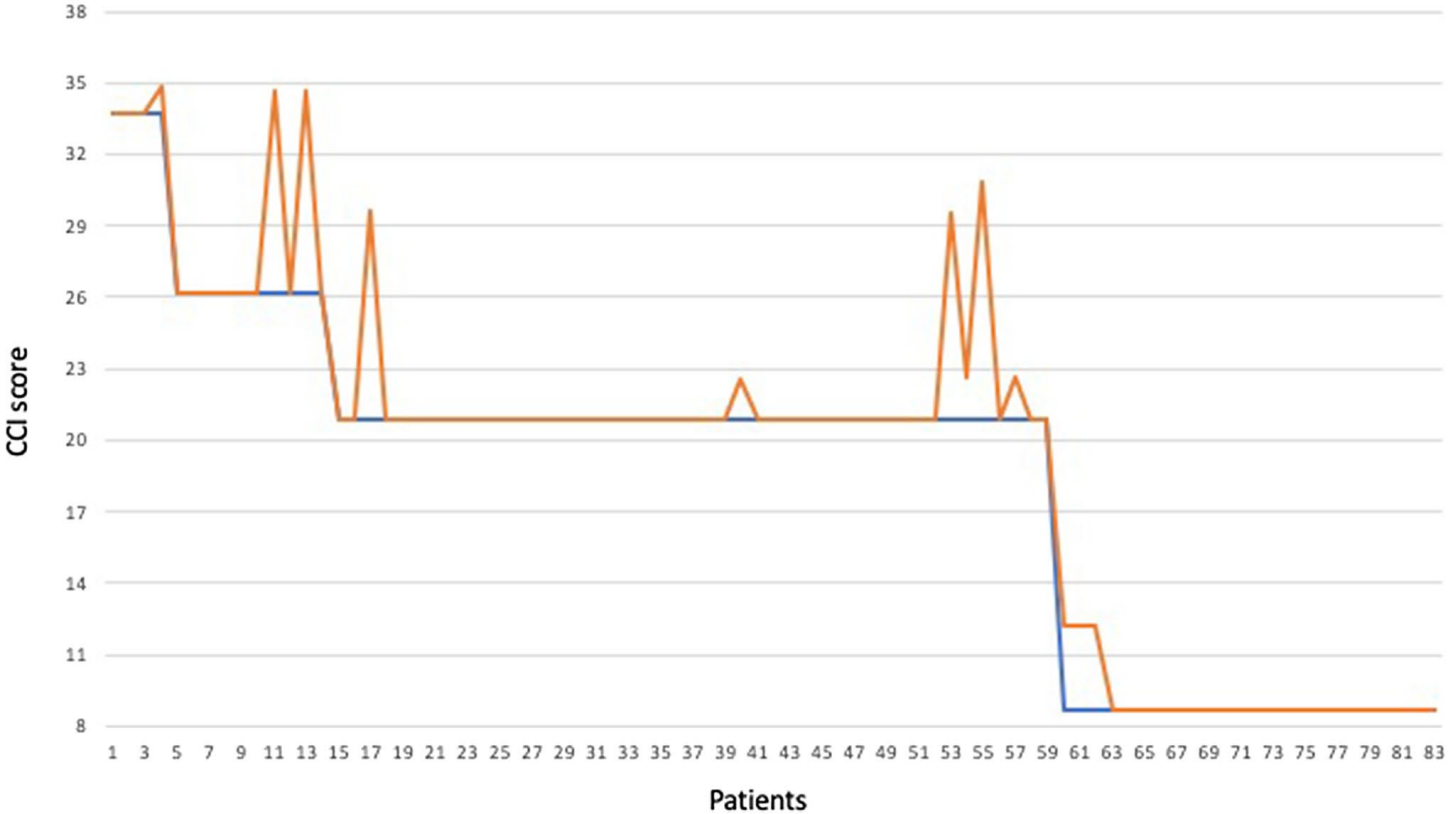
Comparison of the cumulative CCI (orange line) with the non-cumulative CDC (highest grade) for each patient

**Fig. 3:**
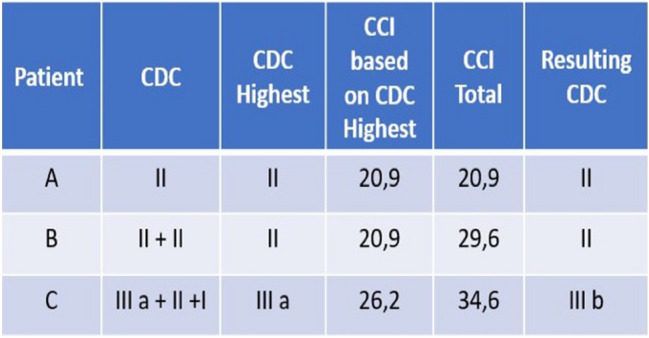
Examples of complications scoring using the CCI and the CDC

Of 83 participants with post-operative adverse events, 12 (4.2%) patients had multiple complications resulting in a higher total CCI score compared to that calculated from the traditional CDC system accounting only for the highest grade [total CCI mean 24.8 (range 12.2–34.8) vs. non-cumulative CDC mean 19.8 (range 8.7–33.7), *p* < 0.01]. Figure 2 plots the cumulative CCI and the non-cumulative CCI based on the highest CDC for each patient on the same graph. Five (1.7%) patients ended up with a CCI score that increased their original CDC grade. Figure 3 reports various examples of complications scoring according to the CCI and CDC. Spearman’s correlation revealed that the CCI enabled for a more accurate prediction of LOS than CDC (CCI: *r* = 0.32; *p* < 0.01 vs. CDC: *r* = 0.26; *p* = 0.01).

Table 2 depicts perioperative characteristics of patients with single vs. multiple complications. Patients with multiple complications had higher stone volume (*p* = 0.02), longer operative time and LOS (all *p* < 0.01) than those with single adverse event. A higher rate of post-operative hospital readmission (33.3% vs. 9.9%, *p* = 0.02) and lower rate of stone free (33.3% vs. 64.7%, *p* = 0.04) were found in patients with multiple complications (Table 2).

**Table 2:** Descriptive statistics of the cohort as segregated according to the number of complications (*n* = 83)

	Single	Multiple	*p* value*
Number of patients [No. (%)]	71 (85.5)	12 (14.5)	
Age (years)			0.6
Median (IQR)	56 (47–63)	57 (50–63)	
Range	19–83	19–84	
Male Gender [No. (%)]	44 (61.9)	9 (75.0)	0.3
BMI (kg/m^2^)			0.7
Median (IQR)	24.8 (21.5–27.6)	23.6 (19.6–28.3)	
Range	17.9–46.1	17.7–35.9	
CHI (score)			0.4
Median (IQR)	0.0 (0.0)	0.0 (0.0)	
Mean (SD)	0.3 (0.2)	0.4 (0.2)	
Range	0–4	0–6	
CHI ≥ 1 [No. (%)]	29 (40.8)	6 (50.0)	0.5
Laterality [No. (%)]			0.8
Right	32 (45.1)	5 (41.7)	
Left	39 (54.9)	7 (58.3)	
Stone volume (cm^3^)			0.02
Median (IQR)	2.5 (1.0–6.9)	6.4 (3.7–15.6)	
Range	0.5–23.5	1.2–26.3	
Multiple stone [No. (%)]	55 (77.4)	10 (83.3)	0.6
Stone density (Hounsfield unit)			0.5
Median (IQR)	1240 (897–1501)	1276 (926–1368)	
Range	400–1900	609–1400	
Multiple access tracts [No. (%)]	16 (22.5)	6 (50.0)	< 0.001
Operative time (min)			< 0.01
Median (IQR)	120 (85–150)	145 (121–186)	
Range	50–234	113–225	
Hemoglobin drop (g/dL)			0.1
Median (IQR)	1.6 (0.8–2.1)	2.1 (0.8–2.7)	
Range	0.0–6.0	0.0–6.0	
Hospitalization time (days)			< 0.01
Median (IQR)	5 (5–8)	11 (6–15)	
Range	3–21	3–22	
Stone-free rate [No. (%)]	46 (64.7)	4 (33.3)	0.04
Readmission [No. (%)]	7 (9.9)	4 (33.3)	0.02

*BMI* body mass index, *CHI* Charlson Comorbidity Index

*p value according to the Mann–Whitney test and Fisher Exact test, as indicated

Table 3 reports univariable and multivariable linear regression models testing potential predictors of LOS. Patient’s BMI, stone volume, multiple access tracts and having one or ≥ 2 complications were all found to be associated with longer LOS (all *p *< 0.01) (Table 3). Multivariable linear regression analysis showed that BMI (beta 0.1; *p* = 0.02), stone volume (beta 0.1; *p* = 0.01) and one (beta 1.5; *p* < 0.001) or multiple complications (beta 6.4; *p* < 0.001) were independent predictors of longer LOS, after accounting for CHI. Of clinical relevance, the average LOS for mPCNL with multiple complications was expected to be 5 days longer than that of surgeries with single adverse event (*p* < 0.001).

**Table 3:** Linear regression models predicting hospitalization time in the whole cohort

	UVA model B; *p* value [95% CI]	MVA model B; *p* value [95% CI]
Age	0.01; 0.3 [− 0.01–0.04]	
BMI	0.13; < 0.01 [0.03–0.22]	0.1; 0.02 [0.01–0.16]
CHI ≥ 1	1.01; 0.04 [0.05–1.95]	0.42; 0.35 [− 0.47–1.31]
Female Gender	− 0.15; 0.3 [− 1.45–0.42]	
Stone Volume	0.19; < 0.001 [0.13–0.26]	0.1; 0.01 [0.02–1.47]
Multiple stones	0.29; 0.01 [0.05–0.54]	
Multiple access tracts	3.01; < 0.001 [1.85–4.17]	
Number of complications		
None	Ref -	Ref
Single	1.99; < 0.001 [0.98–3.01]	1.56; < 0.001 [0.64–2.49]
Multiple	5.87; < 0.001 [3.71–8.05]	6.42; < 0.001 [4.59–8.26]

*UVA* Univariate model, *MVA* Multivariate model, *BMI* body mass index, *CHI* Charlson Comorbidity Index

Table 4 reports univariable logistic regression analysis testing the association between clinical variables and hospital readmission. mPCNL with multiple complications (vs. single) were associated with fivefold higher risk of hospital readmission than those with single complication (*p* = 0.02).

**Table 4:** Logistic regression models predicting hospital readmission

UVA model	OR	*p* value	95% CI
Age	0.91	0.2	0.97–1.02
BMI	1.01	0.9	0.88–1.13
CHI ≥ 1	1.16	0.81	0.32–4.17
Female gender	0.62	0.51	0.15–2.56
Stone volume	1.03	0.37	0.96–1.11
Multiple access tracts	1.01	0.69	0.15–3.72
Multiple complications vs. single	4.57	0.02	1.09–9.13

*UVA* Univariate model, *BMI* body mass index, *CHI* Charlson Comorbidity Index

## Discussion

This is the first study which introduces and validates the CCI in mPCNL cases for kidney stones. We found that the CCI was more accurate than the CDC in depicting the overall burden of post-operative complications and that the CCI enabled for a more precise prediction of LOS than the Clavien classification. Approximately 4.2% of patients showed a higher CCI value than that corresponding to their highest CDC grade, thus suggesting that the CCI is relevant in capturing minor complications which are underestimated by the Clavien–Dindo score. Of note, having multiple complications after mPCNL was associated with longer LOS and higher rates of hospital readmission.

Quality control in urological surgery is crucial to ensure patient safety and improve patient care. Therefore, it is of utmost clinical importance that post-operative complications are reported systematically, objectively and reproducibly [9]. To this aim, the CCI has been introduced to overcome the limitations of the CDC in reporting the overall burden of post-operative complications.

The CCI has been first validated in major uro-oncological series. Vetterlein et al. analyzed a cohort of 506 patients treated with radical cystectomy for bladder cancer and showed that the proportion of cases with severe complications was higher using the CCI than the CDC (31% vs. 11%) [26]. Of note, 99% of patients experienced post-operative complications (any severity) in their series. Likewise, Kowalewski et al., investigated a series of 682 patients treated with radical cystectomy, radical prostatectomy and partial nephrectomy to validate the CCI [16]. Authors found that the CCI resulted in an upgrading in the Clavien classification for 2.4–32.4% of patients. Interestingly, the CCI was more accurate than the CDC in predicting LOS after radical cystectomy [16]. More recently, the CCI was also introduced in endourological procedures. Grüne et al., investigated a cohort of 148 and 179 participants treated with PCNL and URS, respectively, for urinary stones [19]. They revealed that 13.5% and 6.1% of PCNL and URS cases had multiple complications resulting in a higher total CCI score than that based on the highest CDC. The CCI was not associated with LOS in this report [19]. Of note, the PCNL group was highly heterogeneous and included 69 (46.6%) 40 (27%) and 39 (26.4%) patients treated with mPCNL, standard PCNL and combined URS/PCNL, respectively.

Nowadays, the miniaturization of the instruments has improved PCNL outcomes in terms of SFR and post-operative complications, widening the indications of PCNL to a greater range of stone volumes [1]. In fact, mPCNL has become one of the most preferred treatment option by endourologist across Europe [20]. In this study, we specifically aimed to validate the CCI in a series of mPCNL performed at a single tertiary-referral center for stone disease. Our 28.9% overall rate of post-operative complications was lower compared to that from Grüne et al. (41.2%) [19], but probably reflects the lower morbidity associated with smaller access tract of mPCNL as oppose to standard procedures [27]. Similarly, in our cohort, only 4.2% of patients experienced multiple complications. Of clinical relevance, we showed that patients with multiple complications had larger stone volumes, longer operative time and LOS than those with single adverse event. Furthermore, a higher rate of hospital readmission (33.3% vs. 9.9%) and a lower stone-free status (33.3% vs. 64.7%,) were found in patients with multiple complications. Hence, our results suggest that having multiple complications after mPCNL detrimentally impacts on surgical outcomes. The CCI was found to be a better prognosticator of LOS, compared to the CDC, in patients treated with radical cystectomy [16]. This was not noted in a previous cohort of PCNL and URS [19]; however, in the present study, we confirmed that the CCI enabled for a more accurate prediction of LOS than CDC (CCI: *r* = 0.32; *p* < 0.01 vs. CDC: *r* = 0.26; *p* = 0.01). We showed that, along with patient’s BMI and stone volume, having multiple complications was independently associated with longer LOS. Of clinical importance, the average LOS for mPCNL with multiple complications was 5 days longer than that of surgeries with single adverse event. Therefore, in research setting and in clinical practice, the use of CCI, which takes into account multiple adverse events and not only the most severe, gain even more importance in terms of patient’s management and health-related costs [7]. We also found that patients with multiple complications had higher risk of readmission than those with single adverse events. This should be considered by treating physicians when planning patients discharge and follow-up.

This study is innovative because it specifically validates the CCI in a series of mPCNL, which is the most preferred surgical technique for stone diseases by endourologists [20]. Therefore, our study gains a strong characterization in the everyday clinical practice. Conversely, other reports have included mixed populations of standard PCNL, mPCNL and URS/PCNL [19]. The second strength of the study is that we have analyzed a homogenous cohort of patients with a thorough clinical and perioperative evaluation. In particular, only one investigator carried out the retrospective data collection and classification of complications to reduce variability in scoring.

Our study is not devoid of limitations. The retrospective nature of our investigation might lead to data loss in terms of complications; however, as for our internal protocol, patients were checked by phone calls 15 and 30 days post-operatively. Therefore, we can assume that our scoring of complication is rigorous. Also, this was a single center-based study, which raises the possibility of selection biases; thereof, larger studies across different centers and cohorts are needed to externally validate our findings. Lastly, the relatively low rate of complications in our cohort precluded the investigation of association between the CCI and more severe outcomes (e.g., hospital readmission) in a multivariable fashion. For that reason, multicenter studies should further investigate the role of CCI in PCNL.

## Conclusions

In this study, we validated the CCI as a score for reporting post-operative complications after mPCNL. We found that the CCI was more accurate than the CDC in depicting the overall burden of post-operative complications and that the CCI enabled for a more precise prediction of LOS than the Clavien classification. Only 4.2% of patients had multiple complications leading to a higher CCI value than that corresponding to their highest CDC grade. Of note, having multiple complications after mPCNL was associated with longer LOS and higher rates of hospital readmission. Therefore, the CCI is relevant in clinical practice to capture the overall burden of complications, in terms of major and minor adverse events, which have a strong impact in patient’s management and health costs.

## Supplementary Information

Below is the link to the electronic supplementary material.

Supplementary file1 (DOCX 13 KB)
