# Supplementary material for: The comprehensive complication index as a tool for reporting the burden of complications after mini-percutaneous nephrolithotomy: is it time to leave the Clavien–Dindo classification behind?
Source: World J Urol. 2022 May 28;40(7):1829–37. doi: 10.1007/s00345-022-04045-9 (PMC9236985; doi:10.1007/s00345-022-04045-9)
Supplement: Supplementary file 1 — Supplementary file1 (DOCX 13 KB) [file 345_2022_4045_MOESM1_ESM.docx]

**Supplementary Table 1: Detailed characterization of postoperative complications in the whole cohort (n, %)**

Complication type Clavien-Dindo I Clavien-Dindo II Clavien-Dindo IIIa Clavien-Dindo IIIb

Pain 15 (5.2)

Bleeding 6 (2.1) 10 (3.5) 3 (1.0) 3 (1.0)

Infectious 5 (1.7) 39 (13.6) 1 (0.3)

Leakage 3 (1.0)

Drainage 5 (1.7) 5 (1.7)

Renal failure 1 (0.3)

Other 1 (0.3) 1 (0.3) 1 (0.3) 1 (0.3)
